# Surface Energy of Curved Surface Based on Lennard-Jones Potential

**DOI:** 10.3390/nano11030686

**Published:** 2021-03-09

**Authors:** Dan Wang, Zhili Hu, Gang Peng, Yajun Yin

**Affiliations:** 1 Key Laboratory of Mechanics and Control of Mechanical Structures, Interdisciplinary Research Institute, College of Aerospace Engineering, Nanjing University of Aeronautics and Astronautics, Nanjing 211100, China; zhili.hu@nuaa.edu.cn; 2 Department of Engineering Mechanics, School of Aerospace, Tsinghua University, Beijing 100084, China; pg16@mails.tsinghua.edu.cn (G.P.); Yinyj@tsinghua.edu.cn (Y.Y.)

**Keywords:** surface energy, geometrical effect, curvatures, Lennard-Jones potential

## Abstract

Although various phenomena have confirmed that surface geometry has an impact on surface energy at micro/nano scales, determining the surface energy on micro/nano curved surfaces remains a challenge. In this paper, based on Lennard-Jones (L-J) pair potential, we study the geometrical effect on surface energy with the homogenization hypothesis. The surface energy is expressed as a function of local principle curvatures. The accuracy of curvature-based surface energy is confirmed by comparing surface energy on flat surface with experimental results. Furthermore, the surface energy for spherical geometry is investigated and verified by the numerical experiment with errors within 5%. The results show that (i) the surface energy will decrease on a convex surface and increase on a concave surface with the increasing of scales, and tend to the value on flat surface; (ii) the effect of curvatures will be obvious and exceed 5% when spherical radius becomes smaller than 5 nm; (iii) the surface energy varies with curvatures on sinusoidal surfaces, and the normalized surface energy relates with the ratio of wave height to wavelength. The curvature-based surface energy offers new insights into the geometrical and scales effect at micro/nano scales, which provides a theoretical direction for designing NEMS/MEMS.

## 1. Introduction

Surface energy or surface tension plays an important role in determining various natural and industrial phenomena, including surface bulking, phrase separation, welding and solidification, diffusion and transportation, etc. [1,2,3,4]. At micro/nano scales, surfaces are highly curved to achieve a lower energy, leading to a greatly increased ratio of surface area to volume. Surface effects become significant for mechanical properties at small scales, such as effective elastic moduli of nanocomposites, the bending, vibration, and buckling responses of nanosized structural elements [5,6,7]. Hence, much effort has been directed to determine the surface energy by means of experimental methods, dynamical simulations and theoretical modeling over the last few decades. Experiments determining surface tension mainly depend on the Laplace method, which relates the excess pressure at a curved surface with the principal radius of curvature at a point on the surface [8], for example maximum bubble pressure [9] and maximum pressure in a drop [10], sessile drop [11], and pendant drop [12], etc. The tested surface energy values will be varied in a range, which are affected by temperature, pressure as well as experimental errors [13]. Atomic simulations can assist to calculate surface energy due to the development of computing technology, such as classical thermodynamics calculations, molecular dynamics simulations, ab initio calculations, and density functional theory [14]. Vollath et al. used the atomistic approach to study surface energy of nanoparticles and discussed the influence of particle size and structure [15]. The surface energy density on rough metal surfaces is investigated by atomistic calculations, and the simulation results suggest an analytical model to determine the surface energy density of rough surfaces by the surface geometric parameters and the surface energy density of planar surface [16]. Vega et al. estimated the surface tension of water with several models by using the test-area simulation method [17].

Besides experiments and simulations, some models have been proposed to study the surface energy and tension. For example, a unitary approach has been proposed for the calculation of surface energy and surface tension of nanoparticle being in equilibrium with its saturated vapor on both flat and curved surfaces at given temperature [18]. Through extensive theoretical calculations of the surface tension of most of the liquid metals, Aqra et al. used the fraction of broken bonds in liquid metals to calculate surface tension and surface energy [19,20]. An analytical approach is developed to estimate the surface energy density of spherical surfaces through that of planar surfaces using a geometrical analysis and the scaling law [21].

Although many works have been done, determination of surface energy on curved surface remains unsolved, especially at small scales. For example, the testing of surface energy at nano scales will be very difficult considering environmental influence as well as non-negligible errors [22,23]. Experimental methods are hard to measure the surface energy on curved solid surface directly [24,25]. For the atomic simulations, Malfreyt et al. pointed out that the simulated system-sizes has an impact on the calculated surface tension values [26]. Moreover, the uncertainty increases if one questions its dependence on particle size or more complicated geometry. Generally, considerations based on classical thermodynamics lead to the prediction of decreasing values of the surface energy with decreasing particle size [27]. However, the atomistic approach, based either on molecular dynamics simulations or ab initio calculations, generally leads to values with an opposite tendency. Vollath et al. points out that an insufficient definition of the particle size can lead to this result [15]. Besides size effect, calculating surface tension by atomic simulation takes a long time and a large amount of computation. Although some theoretical models have discussed the surface energy and tension on spherical particles using empirical parameters, there is a lack of models for more complicated curved surface.

The present paper is aimed to investigate the effect of surface geometry on surface energy by theoretical modelling. The interaction pair potential between particles is assumed to be Lennard-Jones (L-J) potential, which can describe intermolecular interactions between electronically neutral atoms or molecules [28]. As the L-J potential is a short-range interaction, the surface particles will mainly interact with local particles in the neighborhood. The surface energy is expressed as a function of principle curvatures based on the homogenization hypothesis. The theoretical results for liquid metals are compared with experimental values in the literatures to confirm the reliability. The effect of curvatures are discussed based on spherical particles and compared with numerical experimental results. The geometrical effect on surface energy is investigated on more complicated structures such as sinusoidal surfaces based on curvature-based expression. This work provides a new perspective to understand the geometrical effect on surface energy at small scales, which will provide a direction on the design of MENS/NEMS.

## 2. Models and Methods

L-J potential models the soft repulsive and attractive interactions, which is usually expressed as,

(1) $$U_{LJ}=4\varepsilon \left[{\left(\frac{\sigma }{r}\right)}^{12}-{\left(\frac{\sigma }{r}\right)}^{6}\right],$$

where *r* is the distance between two interacting particles, the depth of the potential well ε is referred to as dispersion energy, and σ is the distance at which the particle-particle potential energy $U_{LJ}$ is zero [28]. For simplification of calculation, a negative exponential potential form $U\left(r\right)=C/r^{n}$ is used to model L-J pair potential. For *n* = 12, $C=4\varepsilon \sigma^{12}$ and *n* = 6, $C=-4\varepsilon \sigma^{6}$, L-J pair potential can be taken as a superposition of negative exponential form taking *n* = 6 and 12.

**Figure 1:**
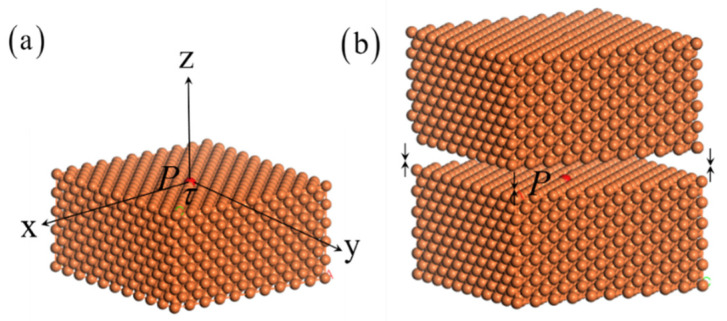
The schematic models: (**a**) The interaction for a particle *P* on the semi-infinite surface body; (**b**) The interaction for a particle *P* inside the body.

As shown in Figure 1, particles are supposed to be hard spheres with the equilibrium radius τ. A homogenization hypothesis is used by assuming particles distributed uniformly in the body with the number density of particles $\rho_{v}$. Then, the interaction potential for a particle *P* on the surface of the semi-infinite flat surface can be expressed as,

(2) $${\bar{U}}_{surf}\left(\tau \right)=\frac{2\pi \rho_{v}C}{\left(n-3\right)\tau^{n-3}}\;\;n\geq 4,$$

In the theory of solid physics, particles on the surface have higher energy than inside particles, as the surface particles interact with less particles [29]. The surface energy and surface tension have different physical meanings. The additional energy for the surface particle compared to inside particles is surface energy [30]. Surface tension is the external energy to form a new unit surface area [31]. For liquids, they are almost the same value, as the atoms can move freely from inside to the surface. For solids, as the surface layer cannot slide freely, the equilibrium for surface tension has to contain the shear stresses between the interiors of the solid and the surface layers, which lead to quite different values of surface energy and surface tension in solids. Here, we use the definition of surface energy to calculate values, as the experimental calculated values from Young’s equation is surface energy.

As shown in Figure 1b, the interaction potential for a particle inside body is twice the value of a surface particle interacted with semi-infinite surface body,

(3) $$U_{inside}\left(\tau \right)=\frac{4\pi \rho_{v}C}{\left(n-3\right)\tau^{n-3}}\;\;n\geq 4,$$

According to the definition of surface energy density, it can be written as the additional energy for a surface particle compared to inside particle per area, i.e.,

(4) $$\bar{\gamma }=\frac{{\bar{U}}_{surf}-U_{inside}}{A}=\frac{2\pi \rho_{v}C}{\left(n-3\right)\tau^{n-3}}\cdot \frac{1}{\pi \tau^{2}}=\;-\frac{2\rho_{v}C}{\left(n-3\right)\tau^{n-1}},$$

**Figure 2:**
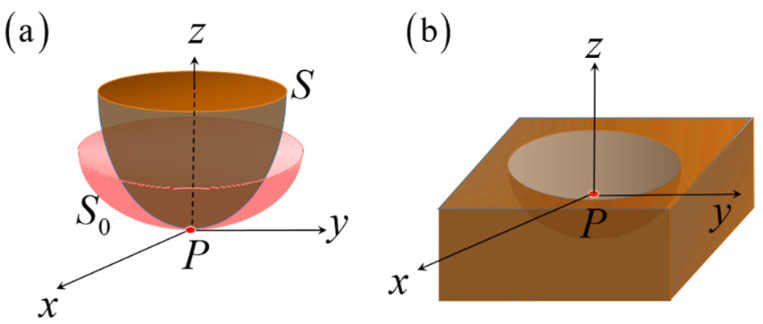
The curved surface: (**a**) the convex surface *S* and approximated surface *S*_0_; (**b**) the concave surface.

Equation (4) is the surface energy density for a particle on a flat surface body. Then we consider the surface energy density on an arbitrary curved surface, as shown in Figure 2. As mentioned above, L-J potential is short-range and decreases fast with the increasing of distance, which means particle only interacts with particles in the nearest zone. A cut-off radius is used when calculating L-J potential in molecular simulations [32]. As shown in Figure 2, coordinate system P−xyz is built with original point at particle *P*. For the uniform of expression, we define that axis *z* points to the concave side of outer surface *S* of curved surface body. Coordinate surface of *x-y* is coincide with tangent surface of *S* at particle *P*, and axes *x* and *y* are along the principle tangent direction of *P* on *S*. In the differential geometry [33], the local shape of a curved surface *S* can be approximated by surface *S*_0_ depicted by two principle curvatures under principle coordinate system *P*-*xyz* [34],

(5) $$z=f(x,y)\approx \frac{1}{2}(c_{1}x^{2}+c_{2}y^{2}),$$

In the previous work, we have expressed the potential of a particle on a curved surface as [35],

(6) $${\overset{⌢}{U}}_{surf}=\frac{2\pi C\rho_{v}}{\left(n-3\right)\tau^{n-3}}\left[1+\frac{n-3}{n-4}\left(\xi \tau H\right)\right],$$

Here, *H* is the mean curvature with the relation between two principle curvatures as,

(7) $$H=\frac{1}{2}\left(c_{1}+c_{2}\right),$$

Substituting Equation (6) into the definition of surface energy Equation (4) leads to the expression of surface energy density on the curved surface,

(8) $$\begin{array}{ll} \bar{\gamma } & =\frac{{\overset{⌢}{U}}_{surf}-U_{inside}}{A}=-\frac{2\pi \rho_{v}C}{\left(n-3\right)\tau^{n-3}}\left[1-\frac{n-3}{n-4}\left(\xi \tau H\right)\right]\cdot \frac{1}{\pi \tau^{2}}\\ & =\;-\frac{2\rho_{v}C}{\left(n-3\right)\tau^{n-1}}\left[1-\frac{n-3}{n-4}\left(\xi \tau H\right)\right] \end{array},$$

ξ is a sign operator with ξ=−1 for convex surface and ξ=1 for concave surface. Equation (8) is the curvature-based surface energy based on L-J pair potential. When the principle curvatures of surface tend to be zero (i.e., *H* = 0), Equation (8) degenerates to the expression of surface energy on flat surface Equation (4). In addition, the value of τH mainly determines the magnitude of curvature effect. As τ is the equilibrium radius of particle and *H* is the mean curvature of curved surface body, τH will be much less than 1 as the curved surface body is consisted of many particles, which means the term inside square brackets of Equation (8) is positive. It is noted that the surface energy of Equation (8) is a local value which is related with the mean curvatures on each points, which means it varies with surface geometry instead of a constant value. Meanwhile, as it is derived from the assumption of hard spheres and L-J potential, surface energy is only related with the hard sphere equilibrium radius τ, the L-J parameters, the number density of hard spheres $\rho_{v}$ and the geometrical effect *H*. For the parameters such as temperature and pressure, their effect will be taken into account by changing these parameters, which will be discussed later.

## 3. Results

### 3.1. The Surface Energy for Liquid Metals on Flat Surface

**Figure 3:**
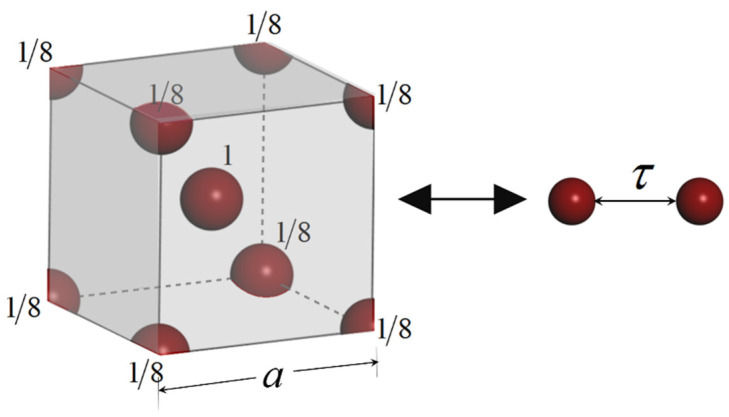
The equivalent atoms in a body-centered crystal (bcc) lattice

**Figure 4:**
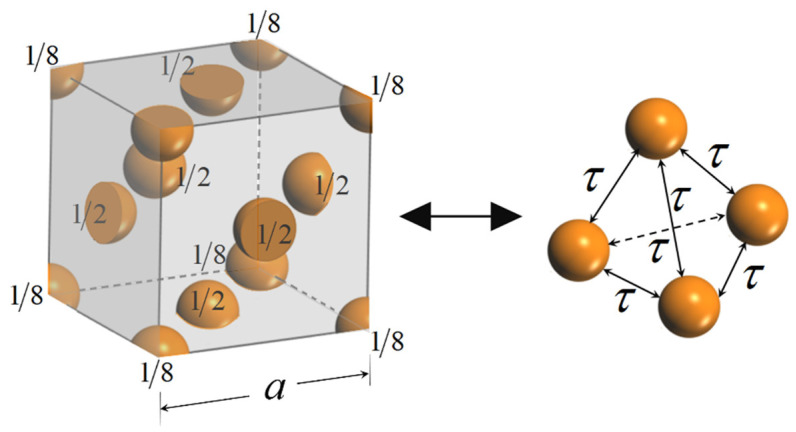
The equivalent atoms in a cubic close-packed (ccp) crystal lattice

In the last section, a model is proposed to calculate the surface energy based on L-J potential. The accuracy of the model (Equation (8)) is verified by comparing the theoretical results on flat surface with experimental results in the literatures. Here, liquid metals are chosen as they are one element liquid with rich experimental values. As Equation (8) is derived based on the assumption that the body is made up with spherical particles homogeneously. The question is how to define equilibrium radius τ for liquid metals. We transfer the crystal structure to a homogeneous model using following assumption. As shown in Figure 3 and Figure 4, taking body-centered crystal (bcc) as an example, there are two full particles in this crystal, the L-J potential between each atom in this crystal is supposed to be equal to the L-J potential between two spherical particles with equilibrium distance τ. Similarly, there are four equivalent atoms within a cubic close-packed (ccp) crystal lattice as shown in Figure 3. The potential relation can be expressed as follows:

(9) $$\sum_{i=1}^{n}\sum_{j=1\left(j\neq i\right)}^{n}4\varepsilon c_{i}c_{j}\left[{\left(\frac{\sigma }{r_{ij}}\right)}^{12}-{\left(\frac{\sigma }{r_{ij}}\right)}^{6}\right]=\sum_{i=1}^{t}\sum_{j=1\left(j\neq i\right)}^{t}4\varepsilon \left[{\left(\frac{\sigma }{\tau }\right)}^{12}-{\left(\frac{\sigma }{\tau }\right)}^{6}\right],$$

where *n* is the total number in a crystal lattice, i.e., *n* = 9 in bcc lattice and *n* = 14 in a ccp lattice. $c_{i}$ and $c_{j}$ is the equivalent ratio for atoms *i* and *j* in a lattice, for example, *c* = 1/8,1/2,1 for atoms on point angle, surface of lattice and inside crystal respectively. *t* is the equivalent atoms number in a crystal lattice, i.e., *t* = 2 for bcc and *t* = 4 for ccp. The number density in Equation (8) is calculated by $\rho_{v}=t/a^{3}$, where *a* is the side length of lattice shown in Figure 3 and Figure 4. For other lattice types, Equation (9) is also adopted to estimate the equilibrium radius τ. It is noted that Equation (9) is an estimation method to define equilibrium radius τ, which considers the L-J potential in the nearest neighbor contributions as crystal are periodic structures. It will be more precious to consider a larger system to define equilibrium radius τ, but here the numerical results have shown an acceptable approximation by considering one lattice. We have listed the crystal type, L-J parameters, number density $\rho_{v}$, the equilibrium radius τ for several metals in Table 1. The abbreviation of crystal type is cubic close-packed (ccp), body-centred cubic (bcc), rhombohedral (rhomb), monoclinic (mo), trigonal (tri), and hexagonal close-packed (hcp).

The surface energy is calculated using Equation (8) and compared with the experimental data in the literature for flat surface in Table 2. The error is defined as,

(10) $$\mathrm{Error}=\frac{\left|\mathrm{Ther}-\mathrm{Exp}\right|}{\mathrm{Exp}}\times 100\%,$$

From Table 2, the curvature based surface energy model is confirmed to be feasible, as the error is within 5% for most cases. As the surface energy varies in a range according to different experimental methods, it is acceptable when the theoretically estimated surface energy is around experimental values.

### 3.2. The Surface Energy for Spherical Surface

**Figure 5:**
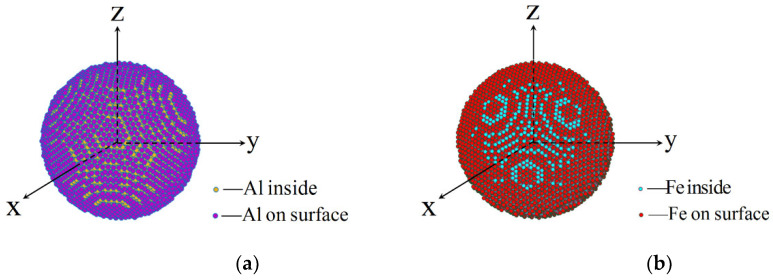
The schematic model of Al and Fe spherical particles: (**a**) The Al particles with surface and inside atoms; (**b**) The Fe particle with surface and inside atoms.

**Figure 6:**
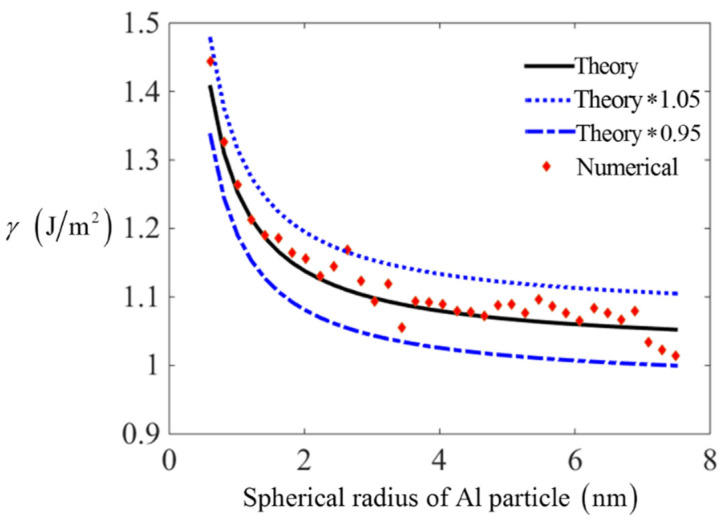
The comparison between theoretical surface energy and numerical integration results for Al spherical particles.

**Figure 7:**
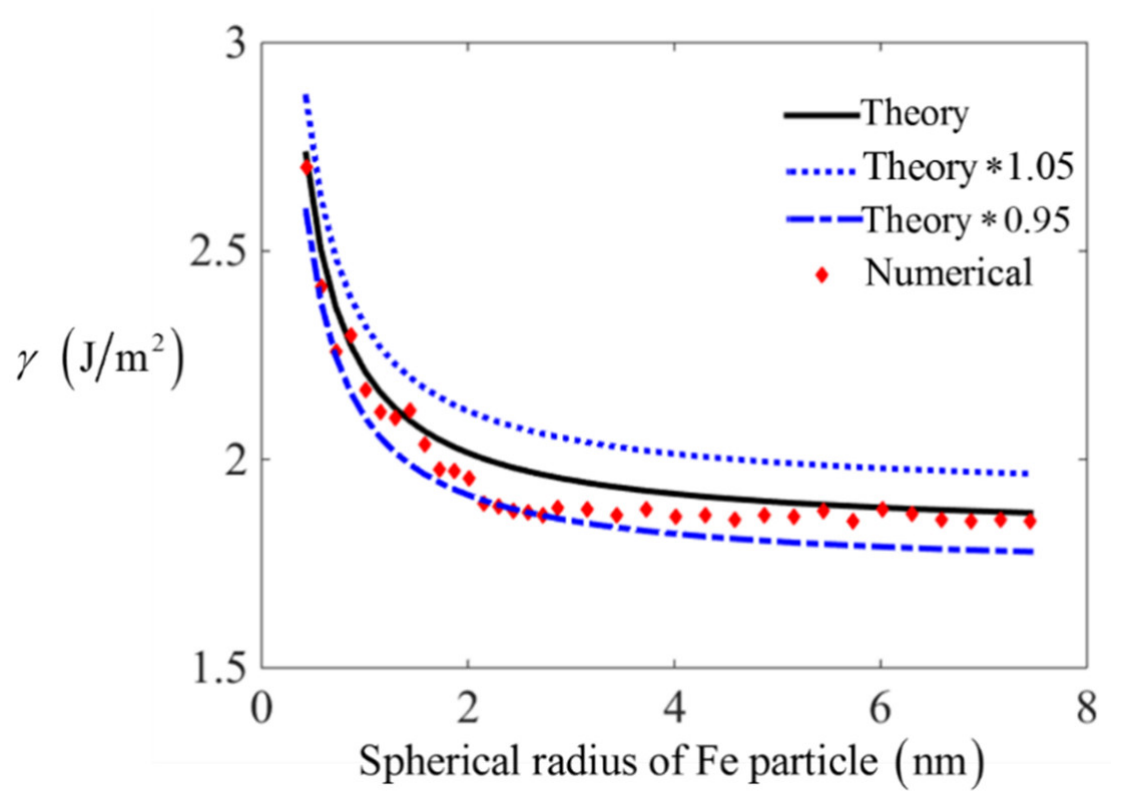
The comparison between theoretical surface energy and numerical integration results for Fe spherical particles.

To test the reliability of curvature-based surface energy (Equation (8)) further, the surface energy on spherical particles is calculated by numerically integration method. As shown in Figure 5, the sphere structures for metals are created by assuming $x^{2}\left(i\right)+y^{2}\left(i\right)+z^{2}\left(i\right)\leq {\left(\eta *a/2\right)}^{2}$, where *a* is the length of lattice, and the radius of sphere is set to η∗a/2, where η is an integer. The surface particles are chosen by satisfying the condition $\left[\left(\eta -1\right)a/2\right]\leq x^{2}\left(i\right)+y^{2}\left(i\right)+z^{2}\left(i\right)\leq {\left(\eta *a/2\right)}^{2}$, supposing that the total number of surface particles is *n*. Based on the L-J pair potential, the surface energy is calculated by adding up the external energy for all surface particles compared to inside particles and then divided by the total surface area $n\pi \tau^{2}$. Here, Fe and Al atoms are chosen to compare the theoretical surface energy with numerical integration results, which are shown in Figure 6 and Figure 7, respectively. The specific calculating errors are put as figures in Appendix A.

From Figure 6 and Figure 7, it is confirmed that the curvature-based surface energy based on L-J potential is in accordance with numerical integration results with an error around 5%. This error is acceptable at nano scales as a result of homogenization hypothesis. The curves in Figure 6 and Figure 7 also indicate that the values of surface energy decrease with the increasing of spherical radius. Here, spheres with larger radius is not considered for two reasons, one is that the curvature effect will be negligible on spheres with larger radius, and another reason is that the estimated error of curvature based surface energy will decrease with the increasing of radius. In addition, the calculation task will also be huge and take a long time, thus the testing error for sphere with larger radius is omitted here.

**Figure 8:**
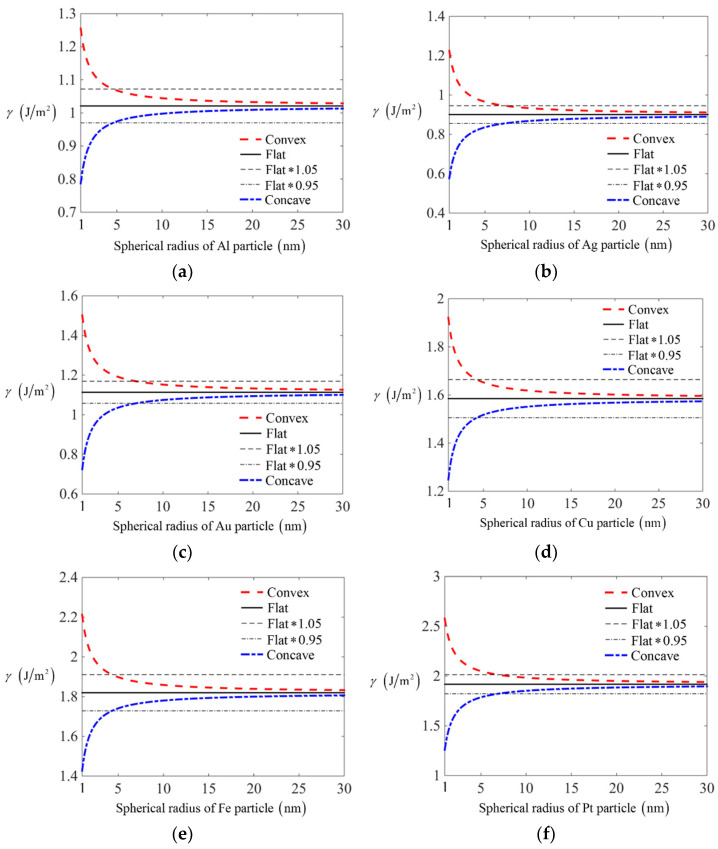
The curvature effect on surface energy for spherical convex and concave surface of different crystals: (**a**) Al particles; (**b**) Ag particles; (**c**) Au particles; (**d**) Cu particles; (**e**) Fe particles; (**f**) Pt particles.

Then we consider several metals with two different surface geometries, i.e., the convex spherical surface and concave spherical surface, the surface energy is shown in Figure 8. It is confirmed that the surface energy will increase with the decreasing of curvatures on concave surface. While for convex surface, the surface energy will decrease with the decreasing of curvatures. Compared to surface energy on a flat surface, the surface energy will increase on a convex surface and decrease on a concave surface with the decreasing of scales. This is consistent with the facts that the surface energy comes from the lack of bonds for surface particles compared to the inside particles. For particles on convex surface, the lacking bonds will increase, leading to surface energy larger than flat surface, and this effect becomes significant on small scales. However, particles on concave surface will have smaller lacking bonds compared to particles on flat surface, which leads to smaller surface energy. From Figure 8, it is also noted that the effect of curvatures on surface energy will exceed 5% compared to one for flat surface when the radius of spherical particles becomes smaller than 5nm. Hence, the curvature effect will be non-negligible when the surface curvatures become larger than 1/5 nm^−1^, which is in consistent with the conclusion mentioned in Ref. [21].

### 3.3. The Curvature Effect on Curved Body with Sine Surface

**Figure 9:**
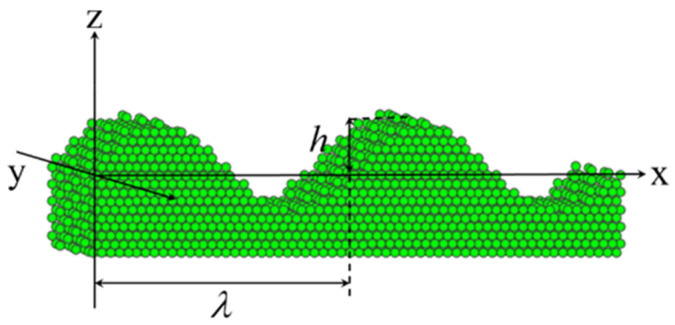
The schematic model of sinusoidal surface.

In this section, a more complex surface geometry with a sine surface will be considered. As shown in Figure 9, the equation of the sinusoidal surface is set to,

(11) $$z=h\sin\left(\frac{2\pi x}{\lambda }\right),$$

where *h* and λ are the amplitude and wavelength of the sinusoidal surface respectively. The principle curvatures of the sinusoidal surface are,

(12) $$c_{1}=0,c_{2}=-\frac{4\pi^{2}h}{\lambda^{2}}\sin\left(\frac{2\pi x}{\lambda }\right)\frac{1}{{\left[1+\frac{4\pi^{2}h^{2}}{\lambda^{2}}\cos^{2}\left(\frac{2\pi x}{\lambda }\right)\right]}^{3/2}},$$

**Figure 10:**
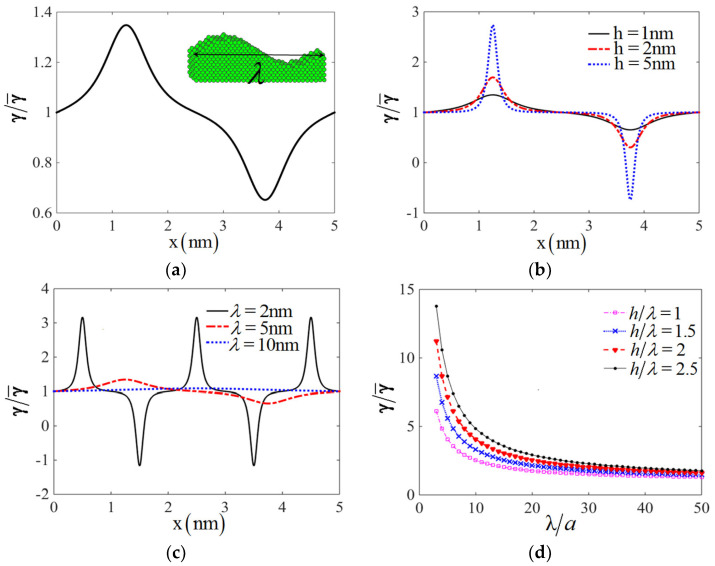
The surface energy on sinusoidal surface: (**a**) The ratio of surface energy on sinusoidal surface to one on flat surface when h=1 nm and λ=5 nm. (**b**) The surface energy distribution on sinusoidal surface with different *h* when λ=5 nm; (**c**) The surface energy distribution on sinusoidal surface with different λ when h=1 nm; (**d**) The normalized surface energy on convex part as a half of sinusoidal periodic surface at different wavelength λ with several ratio h/λ.

The mean curvature is defined as $H=\left(c_{1}+c_{2}\right)/2$. Taking Cu as an example, its crystal type is fcc with lattice constant a = 3.615A. The ratio of surface energy on the sinusoidal surface to one on flat surface is drawn in Figure 10a for h=1 nm and λ=5 nm, which confirms the variation of the surface energy at different positions on sinusoidal surface. The distribution of surface energy shown in Figure 10a is very similar to the one calculated by DFT method in Ref. [16]. The relation between surface energy and wavelength λ as well as amplitude *h* can also be obtained, which are shown in Figure 10b,c respectively. The values of surface energy will increase with the increasing of amplitude *h* and decrease with the increasing of wavelength λ. If we define the normalized surface energy on convex part of sinusoidal period as,

(13) $$\tilde{\gamma }=\frac{2}{\lambda }\int_{0}^{\lambda /2}\gamma \left(x\right)dx,$$

The variation of normalized surface energy compared to flat surface energy with different ratio of wavelength and amplitude is drawn in Figure 10d. The results show the normalized surface energy is larger with higher ratio of wavelength and amplitude, and tends to the surface energy on flat surface when wavelength becomes more than 20 times of lattice constant *a*.

## 4. Discussion

In this section, some discussions towards curvature-based surface energy are presented. Firstly, we want to compare the curvature based surface energy with the Tolman equation which builds the relation between surface energy and spherical radius for nanoparticles [61].

(14) $$\frac{\gamma }{\bar{\gamma }}=\frac{1}{1+\frac{2\delta }{r}}\approx 1-\frac{2\delta }{r},$$

where γ represents the surface energy of spherical particle, $\bar{\gamma }$ is the surface energy for flat surface. δ is the length parameter which is related with the particle’s property, but the physical meaning is unknown.

According to curvature based surface energy, the ratio for spherical surface energy and flat energy is,

(15) $$\frac{\gamma }{\gamma_{0}}=1-\frac{n-1}{n}\frac{\tau \;}{r}\;\;n\geq 4,$$

If we compare Equation (15) with Equation (14), it is interesting that two become the same if assuming $\delta =\frac{n-1}{2n}\tau$, which may give a physical definition for δ.

As mentioned above, it is noted that parameters of temperature and pressure have not appeared in curvature-based surface energy (Equation (8)). However, they can affect the equilibrium radius indirectly. Generally, higher temperature and lower pressure lead to larger equilibrium radius, causing a decrease in surface energy. However, this effect is not that obvious for solids, as the distance between atoms is not easy changed by temperature and pressure.

At last, we want to discuss the application conditions for curvature based surface energy. Firstly, it is derived based on the L-J pair potential between particles, which is a short-range interaction potential. It has not taken the long range interaction such as electrostatic interaction into consideration. However, the geometrical effect of long range interaction on surface energy is very small compared to short range interaction. Secondly, the surface energy is derived based on the model of hard sphere model, which means the determination of sphere equilibrium radius is important to the value of surface energy. It is not easy to determine the equilibrium radius for system with various particles. The equilibrium radius maybe decided based on the known surface energy for a flat surface, and then the geometrical effect for surface energy on a curved surface can be estimated.

## 5. Conclusions

The curvature based surface energy is discussed based on the L-J pair potential. The surface energy for flat surface and spherical particles are compared with results by experimental and numerical integration method to confirm the reliability of the curvature-based surface energy. By considering the surface energy on a spherical convex and concave surface, it is confirmed that the surface energy will increase on the concave surface and decreased on the convex surface with the increasing of scales, which will degenerate to the value on the flat surface. The curvature effect will be obvious for the spherical surface with the curvatures larger than 1/5 nm-1 with an increment more than 5%. The curvature-based surface energy can estimate the surface energy on more complex surface such as sine surface, and provide a physical meaning of the Tolmann length to understand the curvature effect of surface energy for spherical particles.

## Figures and Tables

**Table 1:** The parameters in Equation (8).

Metals	Crystal	ε (J)	$\rho_{v}(/m^{3})$	σ (nm)	τ (nm)	Ref for L-J
Ag	ccp	2.2438 × 10^−19^	5.8464 × 10^28^	0.2648	2.2812	[36]
Au	ccp	2.6426 × 10^−19^	5.8895 × 10^28^	0.2646	0.2841	[37]
Al	ccp	2.3552 × 10^−19^	6.0214 × 10^28^	0.2548	0.2933	[38]
Ni	ccp	4.2299 × 10^−19^	9.1417 × 10^28^	0.2239	0.1915	[39]
Cu	ccp	3.3300 × 10^−19^	8.4678 × 10^28^	0.2297	0.2869	[40]
Pb	ccp	1.2224 × 10^−19^	3.2980 × 10^28^	0.3189	0.2795	[41]
Pd	ccp	2.9760 × 10^−19^	6.7953 × 10^28^	0.2451	0.2133	[42]
Pt	ccp	4.3684 × 10^−19^	6.6192 × 10^28^	0.254	0.2189	[43]
Fe	bcc	4.5210 × 10^−19^	8.4922 × 10^28^	0.2267	0.2804	[40]
Mo	bcc	7.1165 × 10^−19^	6.4183 × 10^28^	0.2489	0.2132	[40]
Cr	bcc	4.3086 × 10^−19^	8.3377 × 10^28^	0.2281	0.2831	[40]
W	bcc	9.2891 × 10^−19^	6.3071 × 10^28^	0.2503	0.2127	[40]
V	bcc	5.5791 × 10^−19^	7.2389 × 10^28^	0.2391	0.2042	[40]
B	rhomb	2.6376 × 10^−19^	2.5641 × 10^28^	0.3543	2.4731	[44]
Bi	mo	1.9520 × 10^−19^	2.8170 × 10^28^	0.059	0.0829	[45]
Sb	tri	3.6896 × 10^−19^	3.1745 × 10^28^	0.354	0.8336	[46]
Zn	hcp	1.0074 × 10^−19^	7.5140 × 10^28^	0.244	0.2217	[47]

**Table 2:** Comparison of surface energy by Equation (8) and experimental data.

Metals	γ in Equation (8) (J/m^2^)	γ in Experiment (J/m^2^)	Ref for γ in Experiment	Error (%)
Ag	0.9006	0.954	[48]	5.6
Au	1.1134	1.1154	[49]	0.18
Al	1.0210	1.1050	[50]	7.60
Ni	1.8464	1.854	[49]	0.41
Cu	1.2966	1.285	[51]	0.90
Pb	0.4390	0.458	[52]	4.15
Pd	1.5391	1.500	[53]	2.61
Pt	1.918	1.865	[54]	2.84
Fe	1.8194	1.820	[55]	0.03
Mo	2.5601	2.510	[56]	1.99
Cr	1.6865	1.700	[55]	0.79
W	2.4828	2.500	[57]	0.69
V	1.9852	1.950	[57]	1.81
B	0.9701	1.060	[58]	8.48
Bi	0.3785	0.379	[55]	0.13
Sb	0.3787	0.380	[59]	0.34
Zn	0.8104	0.816	[60]	0.69

**Figure A1:**
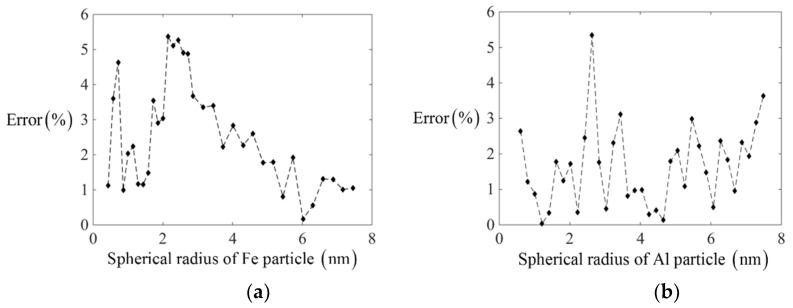
The comparison errors of the theoretical values and numerical results in Figure 6 and Figure 7: (**a**) The errors for spherical Fe particles; (**b**) The errors for spherical Al particles.

## Data Availability

The data presented in this study are available on request from the corresponding author.

## Appendix A

The parameters for the calculation of surface energy on metal particles are listed in Figure A1, with the errors calculated by,

(A1) $$\mathrm{Error}=\frac{\left|\mathrm{Ther}-\mathrm{Exp}\right|}{\mathrm{Exp}}\times 100\%$$

## References

[1] Ehrig S., Schamberger B., Bidan C.M., West A., Jacobi C., Lam K., Kollmannsberger P., Petersen A., Tomancak P., Kommareddy K. (2019). Surface tension determines tissue shape and growth kinetics. Sci. Adv..

[2] Soltani M., Golovin K. (2020). Anisotropy-induced directional self-transportation of low surface tension liquids: A review. RSC Adv..

[3] Pal N., Lee J.-H., Cho E.-B. (2020). Recent Trends in Morphology-Controlled Synthesis and Application of Mesoporous Silica Nanoparticles. Nanomaterials.

[4] Li Z., Yu G., He X., Li S., Tian C., Dong B. (2020). Analysis of surface tension driven flow and solidification behavior in laser linear welding of stainless steel. Opt. Laser Technol..

[5] Duan H., Wang J., Huang Z., Karihaloo B. (2005). Size-dependent effective elastic constants of solids containing nano-inhomogeneities with interface stress. J. Mech. Phys. Solids.

[6] Zaragoza J., Fukuoka S., Kraus M., Thomin J., Asuri P. (2018). Exploring the Role of Nanoparticles in Enhancing Mechanical Properties of Hydrogel Nanocomposites. Nanomaterials.

[7] Ru C.Q. (2009). Size effect of dissipative surface stress on quality factor of microbeams. Appl. Phys. Lett..

[8] Navascues G. (1979). Liquid surfaces: Theory of surface tension. Rep. Prog. Phys..

[9] Christov N.C., Danov K.D., Kralchevsky P.A., Ananthapadmanabhan K.P., Lips A. (2006). Maximum Bubble Pressure Method: Universal Surface Age and Transport Mechanisms in Surfactant Solutions. Langmuir.

[10] Li S., Wang F., Xin J., Xie B., Hu S., Jin H., Zhou F. (2019). Study on effects of particle size and maximum pressure drop on the filtration and pulse-jet cleaning performance of pleated cartridge filter. Process. Saf. Environ. Prot..

[11] Koc M., Bulut R. (2014). Assessment of a Sessile Drop Device and a New Testing Approach Measuring Contact Angles on Aggregates and Asphalt Binders. J. Mater. Civ. Eng..

[12] Saad S.M., Policova Z., Neumann A.W. (2011). Design and accuracy of pendant drop methods for surface tension measurement. Colloids Surfaces A Physicochem. Eng. Asp..

[13] Hemmati-Sarapardeh A., Ayatollahi S., Ghazanfari M.-H., Masihi M. (2014). Experimental Determination of Interfacial Tension and Miscibility of the CO2–Crude Oil System; Temperature, Pressure, and Composition Effects. J. Chem. Eng. Data.

[14] Xie Y., Li J., Peng Z., Yao Y., Chen S. (2020). A first-principle study on the atomic-level mechanism of surface effect in nanoparticles. Mater. Today Commun..

[15] Vollath D., Fischer F.D., Holec D. (2018). Surface energy of nanoparticles–influence of particle size and structure. Beilstein J. Nanotechnol..

[16] Wang J., Bian J.-J., Wang G.-F. (2019). Calculation of surface energy density of rough surface by atomic simulations. Appl. Surf. Sci..

[17] Vega C., De Miguel E. (2007). Surface tension of the most popular models of water by using the test-area simulation method. J. Chem. Phys..

[18] Shebzukhova M., Shebzukhov A. (2011). Surface energy and surface tension of liquid metal nanodrops. EPJ Web Conf..

[19] Aqra F., Ayyad A. (2011). Surface energies of metals in both liquid and solid states. Appl. Surf. Sci..

[20] Aqra F. (2014). Correlations for calculating the surface tension and enthalpies of sublimation of alkali halides. Phys. B Condens. Matter.

[21] Wang J., Bian J., Niu X., Wang G. (2016). A universal method to calculate the surface energy density of spherical surfaces in crystals. Acta Mech. Sin..

[22] Roenbeck M.R., Wei X., Beese A.M., Naraghi M., Furmanchuk A., Paci J.T., Schatz G.C., Espinosa H.D. (2014). In Situ Scanning Electron Microscope Peeling To Quantify Surface Energy between Multiwalled Carbon Nanotubes and Graphene. ACS Nano.

[23] Van Engers C.D., Cousens N.E.A., Babenko V., Britton J., Zappone B., Grobert N., Perkin S. (2017). Direct Measurement of the Surface Energy of Graphene. Nano Lett..

[24] Gunjal P.R., Ranade V., Chaudhari R.V. (2004). Dynamics of drop impact on solid surface: Experiments and VOF simulations. AIChE J..

[25] Tyson W. (1975). Surface energies of solid metals. Can. Met. Q..

[26] Goujon F., Ghoufi A., Malfreyt P. (2018). Size-effects on the surface tension near the critical point: Monte Carlo simulations of the Lennard-Jones fluid. Chem. Phys. Lett..

[27] Fischer F., Waitz T., Vollath D., Simha N. (2008). On the role of surface energy and surface stress in phase-transforming nanoparticles. Prog. Mater. Sci..

[28] Pirani F., Brizi S., Roncaratti L.F., Casavecchia P., Cappelletti D., Vecchiocattivi F. (2008). Beyond the Lennard-Jones model: A simple and accurate potential function probed by high resolution scattering data useful for molecular dynamics simulations. Phys. Chem. Chem. Phys..

[29] Harrison W. (2008). Solid-State Physics: Introduction to the Theory Solid-State Physics: Introduction to the Theory, James D. Patterson and Bernard C. Bailey, Springer, New York, 2007. $99.00 (717 pp.). ISBN 978-3-540-24115-7. Phys. Today.

[30] Packham D. (2003). Surface energy, surface topography and adhesion. Int. J. Adhes. Adhes..

[31] Kondo M., Koshizuka S., Suzuki K., Takimoto M. Surface Tension Model Using Inter-Particle Force in Particle Method. Proceedings of the Volume 2: Fora, Parts A and B; ASME International.

[32] Wang D., Peng G., Yin Y. (2020). The van der Waals potential between arbitrary micro/nano curved surfaces in curvature-based form. Chem. Phys. Lett..

[33] Kühnel W. (2015). Differential Geometry.

[34] Wang D., Yin Y., Zhong Z., Su Z., Hu Z. (2019). Surface evolution caused by curvature driven forces based on natural exponential pair potential. Acta Mech. Sin..

[35] Wang D., Yin Y., Wu J., Wang X., Zhong Z. (2015). Curvature-based interaction potential between a micro/nano curved surface body and a particle on the surface of the body. J. Biol. Phys..

[36] Fournier R. (2001). Theoretical study of the structure of silver clusters. J. Chem. Phys..

[37] Shim J.-H., Lee B.-J., Cho Y.W. (2002). Thermal stability of unsupported gold nanoparticle: A molecular dynamics study. Surf. Sci..

[38] Zhang H., Xia Z. (2000). Molecular dynamics simulation of cluster beam Al deposition on Si (100) substrate. Nucl. Instrum. Methods Phys. Res. Sect. B Beam Interact. Mater. At..

[39] Filippova V.P., Blinova E.N., Shurygina N.A. (2015). Constructing the pair interaction potentials of iron atoms with other metals. Inorg. Mater. Appl. Res..

[40] Filippova V.P., Kunavin S.A., Pugachev M.S. (2015). Calculation of the parameters of the Lennard-Jones potential for pairs of identical atoms based on the properties of solid substances. Inorg. Mater. Appl. Res..

[41] Maghfiroh C.Y., Arkundato A., Misto, Maulina W. (2020). Parameters (σ, ε) of Lennard-Jones for Fe, Ni, Pb for Potential and Cr based on Melting Point Values Using the Molecular Dynamics Method of the Lammps Program. J. Phys. Conf. Ser..

[42] Calvo F., Carré A. (2006). Structural transitions and stabilization of palladium nanoparticles upon hydrogenation. Nanotechnology.

[43] Liem S.Y., Chan K.-Y. (1995). Effective pairwise potential for simulations of adsorbed platinum. Mol. Phys..

[44] Firlej L., Kuchta B., Wexler C., Pfeifer P. (2009). Boron substituted graphene: Energy landscape for hydrogen adsorption. Adsorption.

[45] Arkundato A., Su’Ud Z., Sudarko, Hasan M., Celino M. (2015). Molecular dynamics simulation of corrosion mitigation of iron in lead-bismuth eutectic using nitrogen as corrosion inhibitor. J. Phys. Conf. Ser..

[46] Rajgarhia R.K., Spearot D.E., Saxena A. (2010). Molecular Dynamics Simulations of Dislocation Activity in Single-Crystal and Nanocrystalline Copper Doped with Antimony. Met. Mater. Trans. A.

[47] Guo J.Y., Xu C.X., Gu B.X., Sheng F.Y. (2011). Structure evolution of Zn cluster on graphene for ZnO nanostructure growth. J. Appl. Phys..

[48] Moser Z., Gasior W., Pstrus J., Zakulski W., Ohnuma I., Liu X.J., Inohana Y., Ishida K. (2001). Studies of the Ag-In phase diagram and surface tension measurements. J. Electron. Mater..

[49] Keene B.J., Mills K.C., Brooks R.F. (1985). Surface properties of liquid metals and their effects on weldability. Mater. Sci. Technol..

[50] Sarou-Kanian V., Millot F., Rifflet J.-C. (2003). Surface Tension and Density of Oxygen-Free Liquid Aluminum at High Temperature. Int. J. Thermophys..

[51] Fishman M., Zhuang H.L., Mathew K., Dirschka W., Hennig R.G. (2013). Accuracy of exchange-correlation functionals and effect of solvation on the surface energy of copper. Phys. Rev. B.

[52] Lee J., Shimoda W., Tanaka T. (2004). Surface Tension and its Temperature Coefficient of Liquid Sn-X (X=Ag, Cu) Alloys. Mater. Trans..

[53] Paradis P.-F., Ishikawa T., Yoda S. (2002). Noncontact Measurements of Thermophysical Properties of Molybdenum at High Temperatures. Int. J. Thermophys..

[54] Ishikawa T., Paradis P.-F., Koike N. (2006). Non-contact Thermophysical Property Measurements of Liquid and Supercooled Platinum. Jpn. J. Appl. Phys..

[55] Keene B.J. (1993). Review of data for the surface tension of pure metals. Int. Mater. Rev..

[56] Tyson W., Miller W. (1977). Surface free energies of solid metals: Estimation from liquid surface tension measurements. Surf. Sci..

[57] Kumar R., Grenga H.E. (1976). Surface energy anisotropy of tungsten. Surf. Sci..

[58] Millot F., Rifflet J.C., Sarou-Kanian V., Wille G. (2002). High-Temperature Properties of Liquid Boron from Contactless Techniques. Int. J. Thermophys..

[59] Wang C.-T., Ting C.-C., Kao P.-C., Li S.-R., Chu S.-Y. (2016). Investigation of surface energy, polarity, and electrical and optical characteristics of silver grids deposited via thermal evaporation method. Appl. Surf. Sci..

[60] Goicoechea J., Garcia-Cordovilla C., Louis E., Pamies A. (1992). Surface tension of binary and ternary aluminium alloys of the systems Al-Si-Mg and Al-Zn-Mg. J. Mater. Sci..

[61] Tolman R.C. (1949). The Effect of Droplet Size on Surface Tension. J. Chem. Phys..

